# Tailoring surgical approaches in different cloacal cases

**DOI:** 10.3389/fsurg.2026.1780883

**Published:** 2026-04-07

**Authors:** Ahmed Arafa, Abdelhafeez Mohamed Abdelhafez, Omar N. Abdelhakeem, Ahmed M. Akoula, Ahmed S. Ragab, Ahmed E. Arafat

**Affiliations:** 1 Pediatric Surgery Department, Faculty of Medicine, Cairo University, Giza, Egypt; 2 Pediatric Surgery Department, Faculty of Medicine, Beni Suef University, Beni Suef, Egypt; 3 Pediatric Surgery Department, Faculty of Medicine, Minia University, Minia, Egypt; 4 Pediatric Surgery Department, Faculty of Medicine, Portsaid University, Port Said, Egypt

**Keywords:** anatomical variations, cloacal malformations, long common wall channel or short, surgical management, vaginal replacement

## Abstract

**Aim of the study:**

This study describes surgical approaches for different cases of cloaca tailored to anatomical diversity and severity.

**Methods:**

A retrospective cohort study was conducted at Cairo University Specialized Pediatric Hospitals (2021–2024). Data included imaging findings [ultrasound, magnetic resonance image (MRI), distal loop gram, cloacogram, cystoscopy] and surgical techniques (single-stage or staged). Cases were categorized by vaginal depth and common channel length.

**Results:**

Among 20 patients, management was tailored to anatomy. For vaginal depth >2 cm: 4 cases with long common channels (>3 cm) had one-stage laparoscopic rectal and vaginal pull-through; 6 cases with short channels (<3 cm) underwent posterior sagittal rectal pull-through 2 partial urogenital mobilization (PUM),4 total urogenital mobilization (TUM). For staged procedures: 3 cases with long channels and rectal endings below the coccyx underwent rectal pull-through followed by vaginal pull-through; 5 short-channel cases had laparoscopic rectal pull-through followed by TUM (2) or PUM (3). Vaginal depth <2 cm required colon replacement in 2 cases.

**Conclusion:**

Surgical management of cloacal malformations requires individualized approaches based on anatomical factors.

## Introduction

Cloacal malformations are complex congenital defects involving the anorectal, urogenital, and reproductive systems. These anomalies occur in approximately 1 in 50,000 live female births, and are characterized by the abnormal fusion of the rectum, vagina, and urinary tract into a single common channel, resulting in a single opening instead of the typical three (1, 2). The severity of these malformations varies depending on the length of the common channel, the position of the rectal ending, and the presence of other associated anomalies (3). These variations pose significant challenges for diagnosis and surgical management, making each case unique. Despite advances in surgical techniques, there remains a lack of consensus on the optimal approach for managing cloacal malformations, particularly given the anatomical diversity and the variability in severity across different cases (4). This study aims to describe surgical approaches for different cases of cloaca tailored to anatomical diversity and severity.

### Patients and methods

This retrospective descriptive cohort study was conducted in the Pediatric Surgery Department at our Pediatric Hospitals from 2021 to 2024, Patients with associated anomalies (such as renal, urological, or gynecological anomalies) were not excluded, as these conditions are commonly associated with cloacal malformations. Exclusion was limited to incomplete records or loss to follow-up.

with the aim of describing diverse surgical management strategies for cloacal malformations, considering anatomical variations and clinical severity. Mean age of surgery was 2 years, follow up 4 years postoperative. Data collected from patient records included:

1. Patient Presentation and Initial Assessment:
  - General condition at presentation and associated anomalies.
  - Local examination of the external genitalia, noting the number of openings and presence or absence of abdominal distention.
2. Diagnostic Imaging and Investigations:
  - Review of pre-recorded imaging studies, including abdominal ultrasound, MRI, distal loopogram, cloacogram, and cystoscopy, to assess anatomical variations and severity.
3. Surgical Management Approaches:
  - Documentation of the surgical techniques used, noting variations in approach.
  - Recording of the number of surgical stages planned or performed for each case.

This data was analyzed to assess how different anatomical presentations and severity influenced the choice of surgical technique and staging approach.

## Results

This retrospective study included a total of 20 patients with various anatomical presentations of cloacal malformations, with various associated anomalies, we had 2 cases of vaginal duplications, one case with absent right kidney, one case of vesicoureteral reflux, with different management strategies based on severity and anatomical diversity, we depended on cloacogram, cystoscope, MRI to measure common channel, urethral length, and vaginal depth.

**Figure 1:**
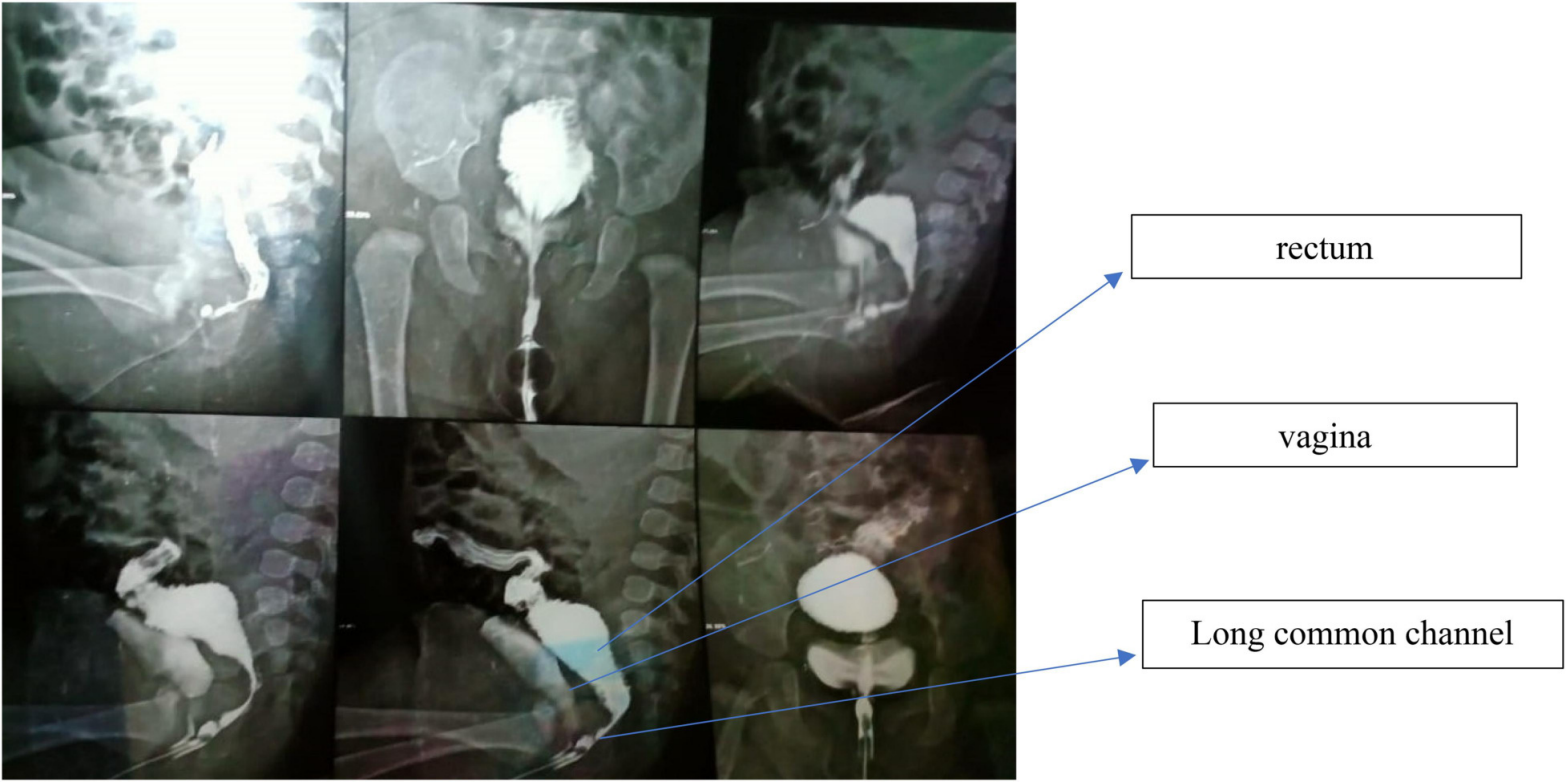
Distal loopogram demonstrating a long common channel and high rectal termination. The rectum (upper arrow) is seen terminating above the level of the coccyx, while the common channel (lower arrow) is elongated (>3 cm). The vagina is identified joining the common channel proximally. These anatomical features supported the decision for a laparoscopic-assisted rectal and vaginal pull-through to allow safe mobilization while minimizing perineal dissection.

**Figure 2:**
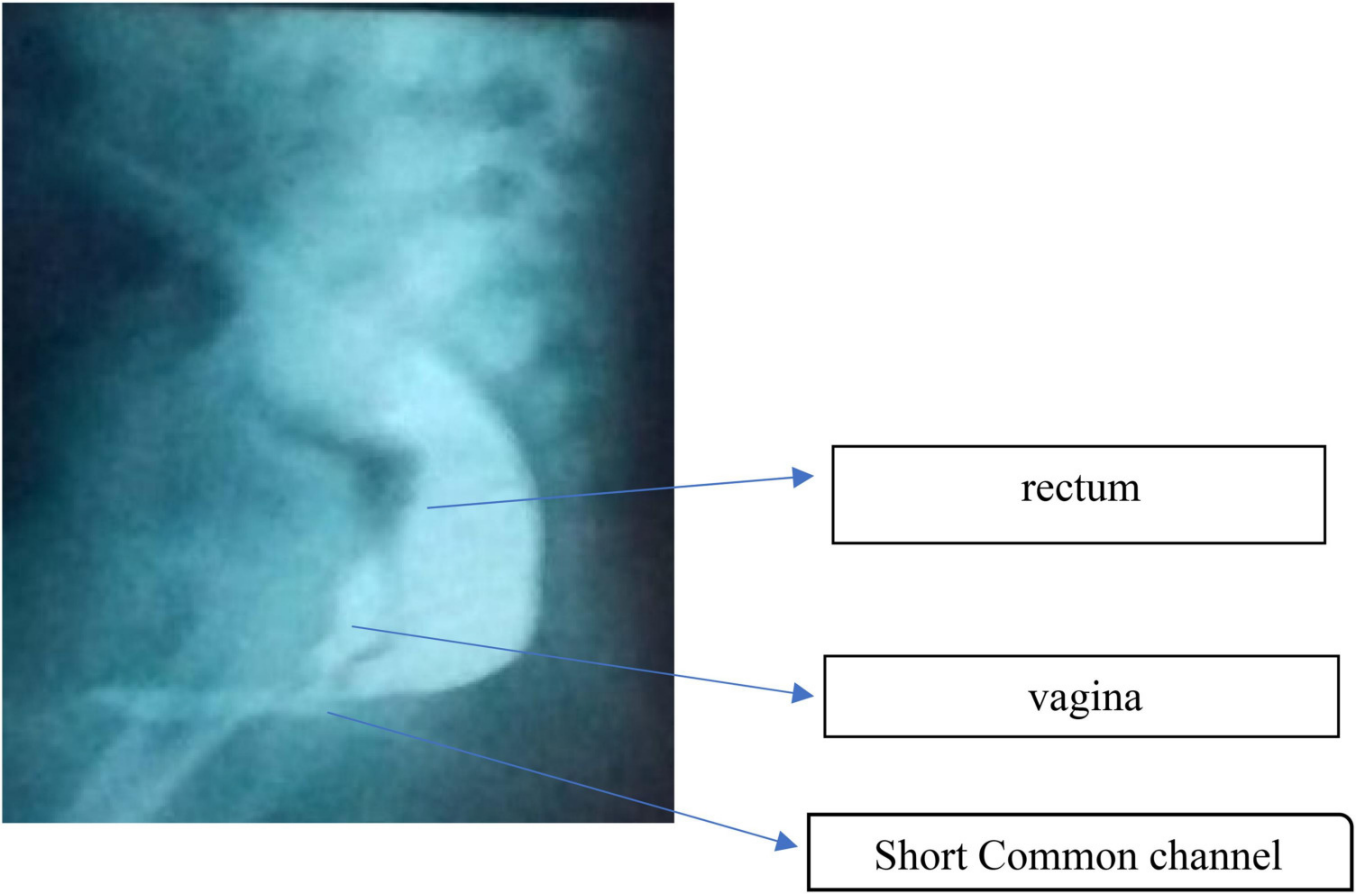
Distal loopogram showing a short common channel with low rectal termination. The rectum is seen terminating below the coccygeal level with a short common channel (<3 cm). The vagina is adequately developed and joins the common channel distally. This anatomical configuration favored a posterior sagittal rectal pull-through combined with partial or total urogenital mobilization in a single-stage procedure.

For cases with a vaginal depth >2 cm:

1. 4 patients with long common channels (>3 cm), urethral length was less than 1.5 cm and rectal endings above the coccyx underwent simultaneous laparoscopic rectal and vaginal pull-through in a single stage (Figure 1).
2. 6 patients with short common channels (<3 cm), urethral length was more than 1.5 cm and rectal endings below the coccyx were treated with rectal pull-through via a posterior sagittal approach, with 2 cases receiving Perineal Urethrovaginal Mobilization (PUM) and 4 cases receiving Total Urogenital Mobilization (TUM) in the same setting (Figure 2).

### In cases requiring a staged approach

**Figure 3:**
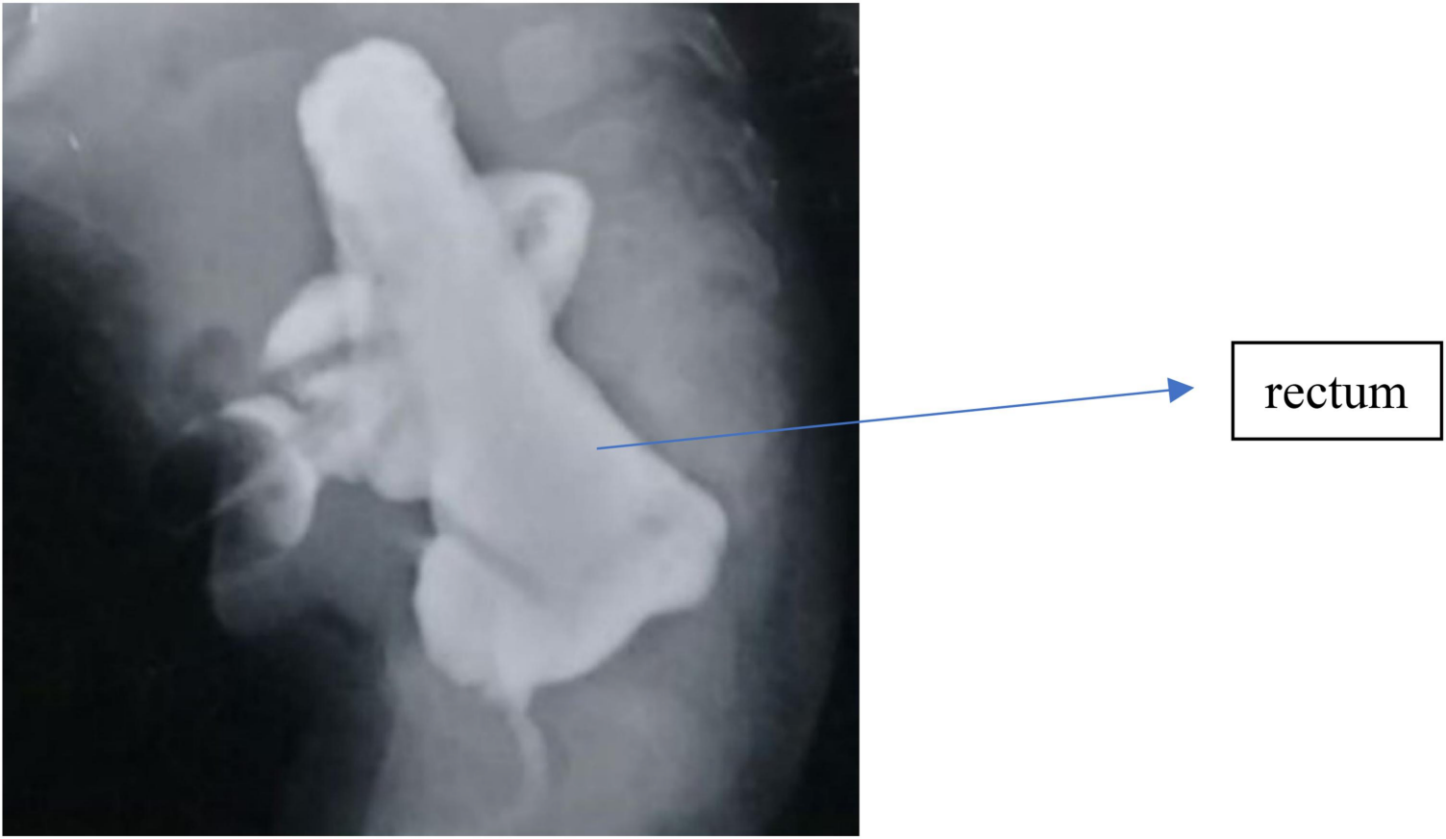
Distal loopogram illustrating a long common channel with unfavorable rectal position. The rectal termination is located above the coccyx, associated with a long common channel. Due to the high rectal position and proximity to the urogenital structures, a staged approach was selected, consisting of laparoscopic rectal pull-through followed by delayed urogenital reconstruction.

Staged approach was done to minimize risk of anesthesia from prolonged time of operation,

1. 3 patients with long common channels (>3 cm), urethral length was less than 1.5 cm and rectal endings below the coccyx initially had rectal pull-through via a posterior sagittal approach, followed by laparoscopic vaginal pull-through after six months.
2. 5 patients with short common channels (<3 cm), urethral length was more than 1.5 cm and rectal endings above the coccyx first underwent laparoscopic rectal pull-through, followed by TUM in 2 cases and PUM in 3 cases six months later. (Figure 3).

**Figure 4:**
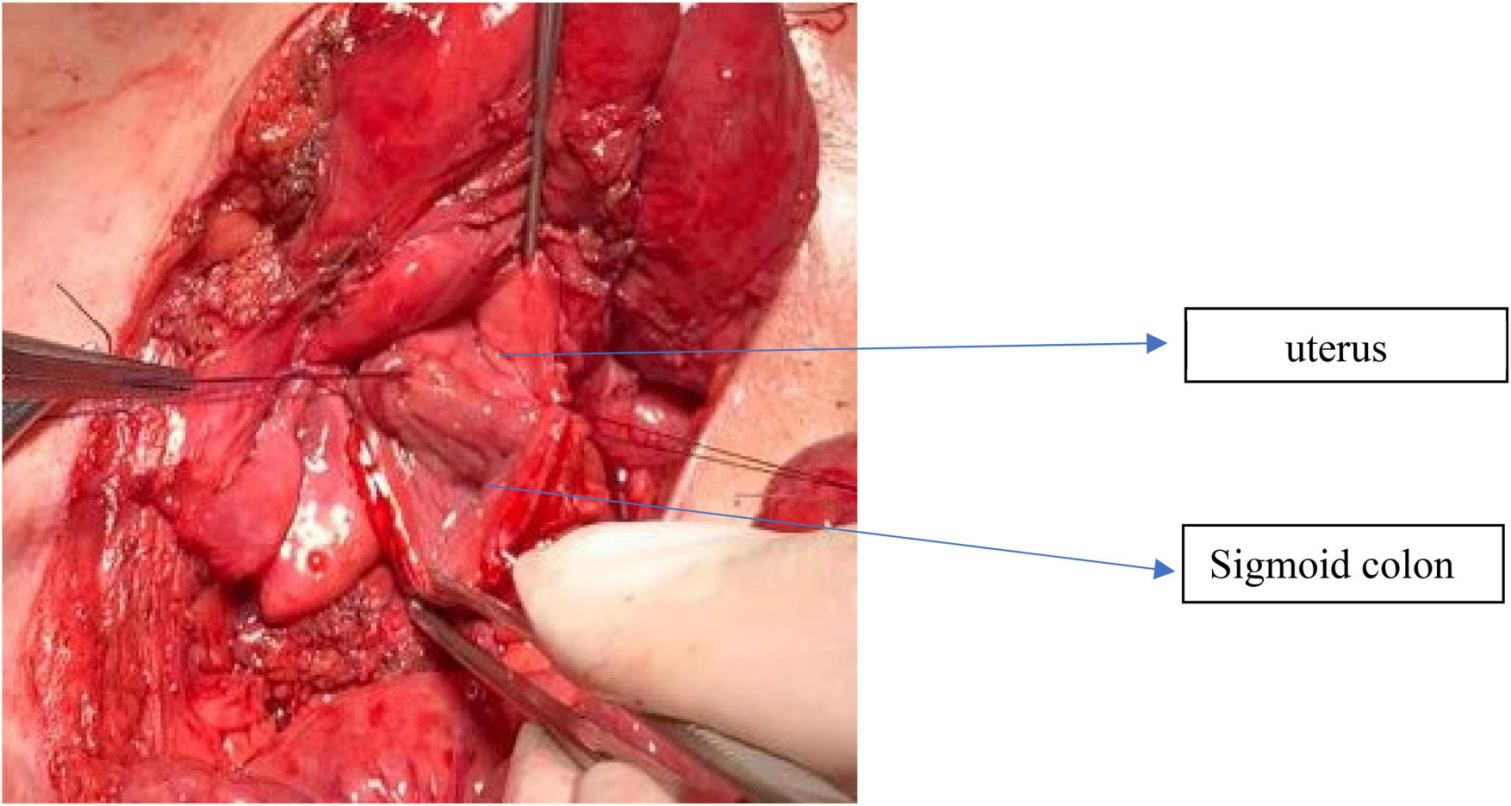
Intraoperative image of vaginal reconstruction using sigmoid colon. The sigmoid colon flap is shown anastomosed to the uterine remnant to create a neovagina in patients with insufficient native vaginal depth (<2 cm). This technique provides adequate length and reliable vascularity, facilitating future functional and cosmetic outcomes.

For 2 patients with vaginal depth <2 cm, colon replacement was performed due to the inadequate vaginal depth (Figure 4).

Immediate postoperative follow-up included (Table 1) anal and vaginal calibration three weeks after surgery, with continued anal dilations as per a structured program. But we still needed long follow up on these cases especially those with long channel to assess urinary incontinence, also in cases with laparoscopic vaginal pull through which we avoided TUM and over dissection around urethral sphincters to minimize risk of urinary incontinence.

**Table 1:** Operative techniques and postoperative outcomes.

Surgical technique	Number of cases *n* (%)	Mean operative time (hours)	Vaginal stricture *n* (%)	Rectal stricture *n* (%)	Urinary retention *n* (%)	Urinary incontinence *n* (%)	Urethrovaginal fistula *n* (%)	Total cases
Total urogenital mobilization (TUM)	6 (30%)	3.0	1 (16.7%)	1 (16.7%)	1 (16.7%)	2 (33.3%)	0 (0%)	6
Partial urogenital mobilization (PUM)	5 (25%)	2.0	3 (60%)	1 (20%)	1 (20%)	0 (0%)	0 (0%)	5
Laparoscopic vaginal pull-through	7 (35%)	2.0	3 (42.9%)	2 (28.6%)	1 (14.3%)	2 (28.6%)	0 (0%)	7
Colon replacement (coloplasty)	2 (10%)	3.0	0 (0%)	0 (0%)	0 (0%)	1 (50%)	1 (50%)	2
Total	20 (100%)	-	7 (35%)	4 (20%)	3 (15%)	5 (25%)	1 (5%)	20

We follow up our cases along 4 years, postoperative. Five patients (25%) developed urinary incontinence; all showed clinical improvement with clean intermittent catheterization during follow-up. All cases of rectal and vaginal stricture responded to a structured dilatation program, with acceptable bowel emptying, We had 3 cases of rectal stricture, 2 of them were occurred after laparoscopic, the other was occurred after posterior sagittal approach, Cases of rectal and vaginal stricture responded to routine dilation.

3 cases of Urine retention, we put catheter through cystoscope for one week, 5 Cases of Urinary incontinence improved on clean intermittent catheterization for months.

Cystoscopy and vaginoscopy before colostomy closure identified urethrovaginal fistulas, which were repaired, with colostomy remaining in place during the repair.

## Discussion

Cloacal malformations represent one of the most complex congenital anomalies encountered in pediatric surgery, owing to the wide spectrum of anatomical variations involving the anorectal, urogenital, and reproductive systems. Their management requires a balance between achieving anatomical reconstruction and preserving long-term bowel, urinary, and genital function. As previously described, classification based on common channel length remains a cornerstone in guiding surgical strategy (3, 5).

While common channel length has traditionally been emphasized as the primary determinant of surgical complexity, our findings suggest that vaginal depth represents an equally critical anatomical factor influencing surgical planning. Peña and Levitt demonstrated that long common channels are associated with higher rates of urinary dysfunction and increased need for complex reconstruction (3, 5). In our series, however, adequate vaginal depth (>2 cm) allowed successful single-stage reconstruction even in selected long-channel cases, highlighting the importance of incorporating vaginal anatomy into preoperative decision-making.

This observation expands upon existing classifications by demonstrating that reliance on common channel length alone may oversimplify surgical planning and underestimate the feasibility of tailored approaches.

Levitt et al. (5) and Peña et al. (6) described that in short common channel, the posterior sagittal approach without abdominal intervention is generally sufficient, offering a simpler surgical route with favorable functional results. This is consistent with our findings in the patients with a short common channel and vaginal depth greater than 2 cm, where the rectal pull-through was performed using the posterior sagittal approach in one-stage surgeries. In these cases, both rectal and vaginal mobilization could be completed successfully in a single setting.

For long common channel, where the common channel length exceeds 3 cm, more complex procedures, including laparotomy, are often required due to the associated anatomical challenges and higher incidence of urological complications. Our findings corroborate these observations, as we observed that in cases with a long common channel and vaginal depth greater than 2 cm, and a rectal ending above the coccyx, laparoscopic rectal and vaginal pull-through could be performed in a single stage. If rectal ending was below the coccyx, staged procedures were required, as seen in our study where rectal pull-through was performed first, followed by laparoscopic vaginal pull-through six months later.

For patients with a short common channel (<3 cm) and sufficient vaginal depth, posterior sagittal rectal pull-through combined with partial or total urogenital mobilization (PUM/TUM) provided satisfactory anatomical and functional outcomes. This finding is consistent with prior studies reporting favorable results using posterior sagittal approaches in anatomically favorable cases (5, 6).

In contrast, long common channel variants (>3 cm) present greater technical challenges due to the short urethral length and proximity of the urogenital confluence to the sphincter mechanism. Wood et al. emphasized that these anatomical features significantly increase the risk of postoperative urinary incontinence (7) In our experience, laparoscopic-assisted mobilization enabled safer dissection in selected long-channel cases by improving visualization and minimizing perineal dissection.

Wood et al. (8) and Levitt et al. (9) also highlight the importance of addressing associated urological issues when dealing with long common channels. This is in line with our study's approach to ensuring proper preoperative evaluation, such as distal loop grams, cystoscopies, and MRI, to assess both the anorectal and urogenital anatomy. Our findings show that careful preoperative imaging can help plan whether a staged or single-stage procedure is best suited for the patient's specific anatomy, minimizing risks like urethrovaginal fistulas or other complications.

The choice between single-stage and staged reconstruction remains a subject of debate. While single-stage repair offers advantages such as reduced psychological burden and fewer hospital admissions, it may increase operative time and surgical trauma in complex cases (10, 11). In our cohort, staged reconstruction was selectively employed in anatomically challenging scenarios, particularly in long common channel cases with unfavorable rectal position, to minimize operative risk and allow safer vaginal reconstruction at a later stage.

This staged approach is supported by Levitt and Peña, who reported reduced complication rates when vaginal reconstruction is delayed in complex cloacal malformations (5, 9). Our findings reinforce the importance of flexible surgical planning tailored to individual anatomical characteristics rather than adherence to a single reconstructive strategy.

Long-term functional outcomes represent a critical measure of success in cloacal reconstruction. In published series, urinary incontinence rates range from 20% to 30%, particularly in patients with long common channels (3, 11). In our study, urinary incontinence occurred in 25% of patients, predominantly among those with long-channel variants. Notably, these cases demonstrated improvement with clean intermittent catheterization, underscoring the role of conservative postoperative management.

Urethrovaginal fistula formation is another recognized complication following extensive urogenital mobilization (7, 8). In our series, fistulas were identified through routine cystoscopy and vaginoscopy prior to colostomy closure and successfully repaired with the colostomy left *in situ*, consistent with recommended management strategies.

Rectal and vaginal strictures were observed in 15% of patients and responded favorably to structured dilation protocols, aligning with outcomes reported in other institutional experiences (1, 4).

When native vaginal tissue is insufficient, vaginal replacement becomes necessary. Several authors have advocated the use of ileum or sigmoid colon, with sigmoid colon offering advantages in terms of vascular reliability, caliber, and ease of mobilization (12, 13). In our cohort, sigmoid colon replacement was reserved for patients with vaginal depth <2 cm and resulted in satisfactory anatomical outcomes without major morbidity, supporting its role as a preferred option in selected cases.

The introduction of laparoscopy has expanded the surgical armamentarium for cloacal reconstruction. Laparoscopic techniques provide enhanced visualization of pelvic anatomy and facilitate precise mobilization while reducing extensive perineal dissection (7) In our experience, laparoscopic-assisted approaches were particularly beneficial in staged reconstructions, allowing preservation of urethral sphincter integrity and potentially reducing the risk of permanent urinary incontinence.

The limitations of this study include its retrospective design and relatively small sample size, reflecting the rarity of cloacal malformations. the young age of our cohort and the intermediate follow-up period, standardized assessment of fecal continence, sexual function, and reproductive outcomes was not feasible. Although a four-year follow-up provides valuable intermediate outcomes, longer-term evaluation into adolescence is required to assess continence, sexual function, and reproductive outcomes. Future prospective, multicenter studies incorporating standardized functional assessments and urodynamic evaluation are warranted to refine evidence-based surgical algorithms.

While significant advances have been made in the management of cloacal malformations, it is clear that a one-size-fits-all approach does not apply. The variability in anatomical presentation requires a tailored treatment plan, where factors such as the common channel length, vaginal depth, and associated urological anomalies must all be carefully considered. Our study highlights the importance of using a combination of single-stage and staged approaches based on these individual anatomical factors.

## Conclusion

This study highlights the importance of individualized surgical approaches for cloacal malformations, based on anatomical factors such as common channel length, urethral length, vaginal depth, and rectal positioning with acceptable intermediate functional outcomes.

## Data Availability

The raw data supporting the conclusions of this article will be made available by the authors, without undue reservation.
